# Quantitative Proteomic Analysis of Duck Ovarian Follicles Infected with Duck Tembusu Virus by Label-Free LC-MS

**DOI:** 10.3389/fmicb.2016.00463

**Published:** 2016-03-31

**Authors:** Kaikai Han, Dongmin Zhao, Yuzhuo Liu, Qingtao Liu, Xinmei Huang, Jing Yang, Fengjiao An, Yin Li

**Affiliations:** ^1^Key Laboratory of Veterinary Biological Engineering and Technology, National Center for Engineering Research of Veterinary Bio-products, Institute of Veterinary Medicine, Ministry of Agriculture, Jiangsu Academy of Agricultural SciencesNanjing, China; ^2^Jiangsu Key Lab of Zoonosis, Jiangsu Co-Innovation Center for Prevention and Control of Important Animal Infectious Diseases and ZoonosesYangzhou, China

**Keywords:** proteomic analysis, duck, Tembusu virus, ovarian follicles, label-free LC-MS

## Abstract

Duck Tembusu virus (DTMUV) is a newly emerging pathogenic flavivirus that has caused massive economic losses to the duck industry in China. DTMUV infection mainly results in significant decreases in egg production in egg-laying ducks within 1–2 weeks post infection. However, information on the comparative protein expression of host tissues in response to DTMUV infection is limited. In the present study, the cellular protein response to DTMUV infection in duck ovarian follicles was analyzed using nano-flow high-performance liquid chromatography-electrospray tandem mass spectrometry. Quantitative proteomic analysis revealed 131 differentially expressed proteins, among which 53 were up regulated and 78 were down regulated. The identified proteins were involved in the regulation of essential processes such as cellular structure and integrity, RNA processing, protein biosynthesis and modification, vesicle transport, signal transduction, and mitochondrial pathway. Some selected proteins that were found to be regulated in DTMUV-infected tissues were screened by quantitative real-time PCR to examine their regulation at the transcriptional level, western blot analysis was used to validate the changes of some selected proteins on translational level. To our knowledge, this study is the first to analyze the proteomic changes in duck ovarian follicles following DTMUV infection. The protein-related information obtained in this study may be useful to understand the host response to DTMUV infection and the inherent mechanism of DTMUV replication and pathogenicity.

## Introduction

Since April 2010, a severe duck disease outbreak has emerged throughout the major duck-producing regions of eastern China. In addition to ducks, the disease has also affected geese, chickens, and sparrows. The infected ducks exhibited high fever, diarrhea, and other consistent signs including acute anorexia, antisocial behavior, rhinorrhea, ataxia, and paralysis (Su et al., 2011). Necropsy of the infected ducks consistently displayed severe ovarian hemorrhage, ovaritis, and regression. In addition, ruptured ovarian follicles and peritonitis were also found in some infected ducks, whereas enlarged spleen and leg muscle hemorrhage were occasionally noted. The infected egg-laying ducks presented a significant reduction in egg production from 20 to 60%, and even up to 90% in some reported cases, within 1–2 weeks post-infection (Yu et al., 2013), thus causing a severe impact on poultry production and heavy economic losses in China.

Based on the clinical signs and pathological features, this disease was initially designated as duck hemorrhagic ovaritis (DHO). However, further laboratorial studies proved that the causative agent isolated from ducks and geese is Tembusu virus (TMUV), which is a member of the Ntaya virus (NTAV) group belonging to the family *Flaviviridae*, genus Flavivirus (Cao et al., 2011). Similar to other flaviviruses, TMUV is a single-stranded positive-sense RNA virus, with a genome of approximately 10.5 kb and a single open reading frame encoding a large polyprotein (Su et al., 2011). The polyprotein can be cleaved by viral and cellular proteases into three structural proteins (capsid [C]; membrane [M], and envelope [E]) and seven nonstructural proteins (NS1, NS2a, NS2b, NS3, NS4a, NS4b, and NS5; Sun et al., 2014). Nevertheless, to date, information about host cell responses to DTMUV infection is limited.

To understand the pathogenesis of viral infection, knowledge about virus-virus and virus-host interactions is critical. A virus infection may produce dramatic effects on the host cell morphology, transcription and translation patterns, cytoskeleton, cell cycle, innate immune response of the host, and apoptosis pathways, and may also cause inflammation and alter stress responses (Zheng J. et al., 2011). As a result, many functional and morphological changes in the host cells are associated with significant changes in the patterns of expression of host cell genes. Therefore, information on proteome changes in the host following DTMUV infection may be crucial to understand the host response to the virus and viral pathogenesis. In recent times, comparative proteomic analysis has emerged as a valuable tool for the establishment of global host protein profile in response to virus infection. This technique has been used to investigate the proteome changes in cells infected in vitro with classical swine fever virus (Li et al., 2010), infectious bursal disease virus (Zheng et al., 2008), and porcine circovirus (Liu et al., 2014). In addition, this analysis has also been widely employed to examine the mechanisms of viral infection through comparative investigation of the proteome changes in the host tissue in response to infection *in vivo*, such as Marek's disease virus (Lu et al., 2010) and infectious bronchitis virus (Cao et al., 2012).

The DTMUV genome encodes 10 proteins that facilitate exploitation of the host cell machinery to complete the infection cycle and generate viral progeny. However, to the best of our knowledge, there are no reports on the changes in the host tissues in response to DTMUV infection. Thus, the knowledge on proteins involved in these cellular responses to DTMUV infection and the directional changes in protein expression is limited. Label-free proteomic analysis based on nano-flow high-performance liquid chromatography (HPLC)-electrospray tandem mass spectrometry (LC-MS/MS) is emerging as a powerful methodology to determine disease-specific targets. When compared with conventional proteomic technologies, the major advantage of this technique is sensitivity, with thousands of proteins directly identified in typical analyses. In addition, low-abundance proteins can be detected and quantitative information can be acquired from the spectral counts obtained for each peptide sequence (Kikuchi et al., 2012). In this study, we present the results of a label-free quantitative proteomic comparison of the proteome of DTMUV-infected versus mock-infected duck ovarian follicles. Analysis of the obtained data revealed major changes in the proteins involved in central cellular signaling, metabolic pathways, and immune responses. Some individual proteins that were found to be regulated were further validated using qRT-PCR and immunoblotting techniques.

## Materials and methods

### Experimental animals and virus infection

Healthy 180-day-old shelducks were obtained from Siji Poultry Co., Ltd (Jintan, PR China). The ducks were maintained in special cages, and food and water were provided regularly. All animal experiments were conducted in accordance with the regulations and guidelines of animal experimentation outlined by the People's Government of Jiangsu Province. The ducks infected with Tembusu virus JS804 strain were maintained in our laboratory.

### Experimental design

A total of 24 180-day-old shelducks were randomly divided into two groups and housed in different rooms. One group (*n* = 12) was inoculated intranasally with 0.4 mL × 10^5.0^ ELD_50_/mL of the challenge virus, additionally, the other group (*n* = 12) was mock-infected with sterile PBS in the same manner. The two groups of ducks were housed separately in different rooms and observed daily for 10 days post inoculation (dpi) for disease symptoms. At 3, 5, 7, and 10 dpi, three ducks were randomly selected from each group and euthanized using CO_2_ inhalation. The whole ovarian follicles were rapidly separated and washed with ice-cold phosphate buffered saline (PBS). At necropsy, one portion of the ovarian follicles was collected, snap-frozen in liquid nitrogen, and maintained at –80°C for subsequent use in two-dimensional gel electrophoresis and western blot analysis, whereas the other portion was utilized for RNA extraction using Axygen Total RNA extraction Kit (Axygen Biosciences, China).

### Protein sample preparation

The ovarian follicles derived from DTMUV- and mock-infected ducks were washed thrice with ice-cold PBS and lysed using a lysis buffer containing 8 M urea, 2 M thiourea, 4% CHAPS, and 30mM Tris-HCl at 18°C for 15min. Subsequently, the tissues and cells were disrupted by ultrasonication performed for 20 times with 5s pulse on and 5s pulse down at 30% amplitude. The homogenates obtained were centrifuged at 14,000g and 4°C for 20 min, and the supernatants were collected and stored in single-use aliquots at –80°C until use. The protein concentrations in the supernatants were determined using BCA assay (Sigma, USA).

### Gel-assisted digestion

For in-gel digestion, 10 mM dithiothreitol (DTT) was added to each sample and incubated for 1 h at 37°C to reduce the cysteine side chains. Then, 20 mM iodoacetamide was added to the samples and incubated for 30 min in dark at room temperature to alkylate the cysteine side chains. The samples were subsequently diluted six fold with 25 mM ammonium bicarbonate to reduce the urea concentration to 1 M, and then 2% (w/w) modified trypsin (Sigma, USA) was added. The pH was adjusted to 8.0 with 250 mM ammonium bicarbonate, and the samples were incubated for 16 h at 37°C. The digestion efficiency was determined by LC-MS/MS of the digests (aliquots containing 1 μg of the initial amount of protein) desalted by using C18 Cartridge (Sigma, USA) following manufacturer's instructions.

### LC-MS/MS

The protein samples were analyzed by nano-flow high-performance liquid chromatography (HPLC)—electrospray tandem mass spectrometry (LC-MS/MS). The digests were separated by nano-flow liquid chromatography using a SC200 traps 150 μm × 100 mm RP-C_18_ Thermo EASY column (Thermo, USA) at a flow rate of 300 nL/min. The mobile phase A comprised 2% acetonitrile and 0.1% formic acid in water and mobile phase B consisted of 0.1% formic acid in 84% acetonitrile. Following equilibration of the column in 100% solvent A, an aliquot of each digest (10 μL corresponding to 5 μg of total protein) was injected. Subsequently, the organic content of the mobile phase was linearly increased to 45% over 100 min, and then to 100% over 108 min, maintained at 100% over 120 min. The column effluent was directed to a nanospray ion source and attached to a hybrid linear ion trap-Orbitrap mass spectrometer (Thermo Scientific, USA). The peptides were analyzed in positive ion mode and information-dependent acquisition mode to automatically switch between MS and MS/MS acquisition. The MS data were obtained using a data-dependent top 10 method, which dynamically selected the most abundant precursor ions from the survey scan (300–1800 m/z) for higher-energy collisional dissociation (HCD) fragmentation. The target value was determined based on predictive automatic gain control (pAGC), and the dynamic exclusion duration was 25 s. Survey scans were acquired at a resolution of 70,000 at m/z 200, whereas the resolution for HCD spectra was set to 17,500 at m/z 200. The normalized collision energy was 30 eV and the underfill ratio, which specifies the minimum percentage of the target value likely to be reached at maximum fill time, was defined as 0.1%. The instrument was run with the peptide recognition mode enabled. The MS experiments were performed in triplicates for each sample.

### Data analysis

The MS data were analyzed using MaxQuant software (version 1.3.0.5). The peptides and related proteins were searched against the UniProt *Anas platyrhynchos* database (32,619 total entries, downloaded 02/09/15). The initial search was set at a precursor mass window of 6 ppm. The search followed an enzymatic cleavage rule of Trypsin/P and allowed a maximum of two missed cleavage sites and a mass tolerance of 20 ppm for fragment ions. Carbamidomethylation of cysteines was defined as fixed modification, while protein N-terminal acetylation and methionine oxidation were defined as variable modifications. The cutoff of global false discovery rate (FDR) for peptide and protein identification was set to 0.01. The identified peptides were subjected to intensity-based absolute quantification (iBAQ) in MaxQuant to quantify protein abundance. The protein abundance was calculated based on the normalized spectral protein intensity (LFQ intensity).

The sequence data of the selected differentially expressed proteins were retrieved in batches from UniProtKB database (Release 2015_06) in FASTA format. The retrieved sequences were locally searched against SwissProt database (duck) using the NCBI BLAST+ client software (ncbi-blast-2.2.28+-win32.exe) to find the homologous sequences from which the functional annotation could be assigned to the studied sequences. The top 10 blast hits with an *E*-value of less than 1e-3 for each query sequence were retrieved and loaded into Blast2GO2 (Version 2.8.0) for GO mapping and annotation. Following annotation and annotation augmentation steps, the studied proteins were also compared by BLAST against KEGG (Kyoto Encyclopedia of Genes and Genomes) GENES to retrieve their KOs and were subsequently mapped to pathways in KEGG.

### Western blot analysis

To further verify the variation in the differentially expressed proteins identified by the proteomic approaches, 2-5-oligoadenylate synthase-like protein 2 (OASL2), interferon-induced protein with tetratricopeptide repeats 5 (IFIT5), histone deacetylase 2 (HDAC2), thrombospondin-1 (TPP1), and basement membrane-specific heparan sulfate proteoglycan (HSPG) were selected for western blot analysis. The analysis was performed as described previously (Chen et al., 2014) with minor modifications. Briefly, the protein samples (50 μg/lane) were separated using 12% SDS-PAGE gels and transferred to a nitrocellulose transfer membrane. After blocking with 5% (w/v) skimmed milk in TBST (50 mM Tris, pH 8.0, 150 mM NaCl, 0.1% [v/v] Tween-20) for 1 h at 37°C, the membranes were incubated overnight at 4°C with primary rabbit polyclonal antibodies of anti-OASL2 (Abcam, Cambridge, UK), anti-IFIT5 (OriGene, China), anti-TPP1 (Santa Cruz Biotechnology, CA), anti-HSPG (Santa Cruz Biotechnology, CA), mouse polyclonal antibodies of anti-HDAC2 (Santa Cruz Biotechnology, CA) at a dilution of 1:1000. After washing with TBST three times, the membranes were further incubated for 1 h with horseradish peroxidase-conjugated goat anti-rabbit secondary antibody or goat anti-mouse secondary antibody (BOSTER, Wuhan, China) at a dilution of 1:5000. The immunoreactive protein bands were detected using 3,3′-diaminobenzidine (DAB; BOSTER, Wuhan, China), with β-actin as the loading control.

### Real-time RT-PCR

The total RNA was extracted from the ovarian follicles using Axygen Total RNA Extraction Kit (Axygen Biosciences, China) according to the manufacturer's instructions. Synthesis of cDNA was performed with 1 μg of the extracted RNA using a RevertAid™ First Strand cDNA Synthesis Kit (Fermentas, USA), according to the manufacturer's protocol. Specific primers for amplifying various target genes of the MS-identified proteins were designed according to the available gene sequences deposited in GenBank using Lasergene sequence analysis software (DNAStar, Inc., Madison, WI, USA; Table 2). Real-time RT-PCR was performed using SYBR Premix Ex Taq™ II Kit (Takara, China) on the LightCycler 480 real-time PCR system (Roche) as follows: 30 s at 95°C, 40 cycles of denaturation at 95°C for 10 s, and annealing and extension at 55°C for 45 s. The melting curves were obtained and quantitative analysis of the data was performed using a relative quantification (2^−ΔΔCT^) study model. The mock-infected ovarian follicles were used as control (relative expression = 1) and β-actin was employed as an internal reference gene. Each sample was amplified in triplicate.

## Results

### DTMUV infection and clinical signs

From 3 to 6 dpi, transient depression and decreased food intake were observed in DTMUV-infected ducks. Furthermore, a lack of increase in body weight, greenish diarrhea, keratitis, and tearing were noted in the infected ducks. With the disease progression, some ducks exhibited abnormal gait and were unable to stand steadily and kept falling. Contrastingly, the mock-infected ducks remained healthy. The gross lesions of the sacrificed DTMUV-infected ducks mainly comprised severe ovarian hemorrhage, ovaritis, and regression consistently. In addition, ruptured ovarian follicles and peritonitis were also found. Although the viral RNAs were readily detected in the ovaries of the DTMUV-infected birds sacrificed on 3 and 5 dpi, they were not observed on 7 and 10 dpi (Table 1). In contrast, no viral RNAs were detected in the mock-infected group. These results indicated that the ducks in the DTMUV-infected group were successfully infected with Tembusu virus.

**Table 1:** **Virus detection by an E-specific RT-PCR method in ducks infected by DTMUV**.

**Days post-infection**	Virus detection in ovary^*^
3	3/3
5	3/3
7	0/3
10	0/3

* *Shown is the number of positive tissue from ducks/all three tissues from ducks tested in this timing*.

**Table 2:** **Primers for qRT-CR analysis of 10 differentially expressed genes**.

**Gene symbol**	**Gene accession NO**.	**Forward primer sequence (5′-3′)**	**Reverse primer sequence (5′-3′)**	**Amplicon size (bp)**
MGST3	XM_005028171.2	GAGCACGGGCGTATATTCAACTG	AGTGGCTAGGAAGAACAGGAAGG	87
THBS1	XM_005009791.2	AGACACTGATTCTGACCGCATAGG	CTTGATTGGCATTGGGCACATAGG	113
STX7	XM_005009571.2	GGTATCTGGTGGTGCTCCTGAAG	CTTGTGCTTGAGGTTGAGTGTCC	77
NDVFA	XM_005029536.2	CTGGAGGTGCCTGAGGTGAG	ACTGCGAGCCGTAGTGGTG	70
TPP1	XM_013108771.1	CCTGGCTGCTGCTGCTC	GGCTGTCCTCCTCATCATCC	81
SDHB	XM_013187868.1	CGAAACAGGGCAAGGAGCAG	GCAGCAGGCACACAGGATG	89
IRF3	KB742884.1	CAACTCCAAGAACAGCGACGAC	CCTGAGGTGACGGCGAAGAC	51
IFIT5	KF956064.1	ACACTGTTGTTATGGCACTACTTG	AGCAGCATATCGCAGGAAATAAGG	128
VLDL	ABJ16558.1	GAGAGGGACCGTCGTGACTG	CATCCGCCAAGAACTGACCAAC	106
HSPG	XM_013107098.1	CCACCAACGCCTCCTTCCAG	GACAAATCCACCTTCTTCCCATTG	67

### Comparison of differential protein expression in the ovarian follicles between DTMUV- and mock-infected ducks

**Figure 1:**
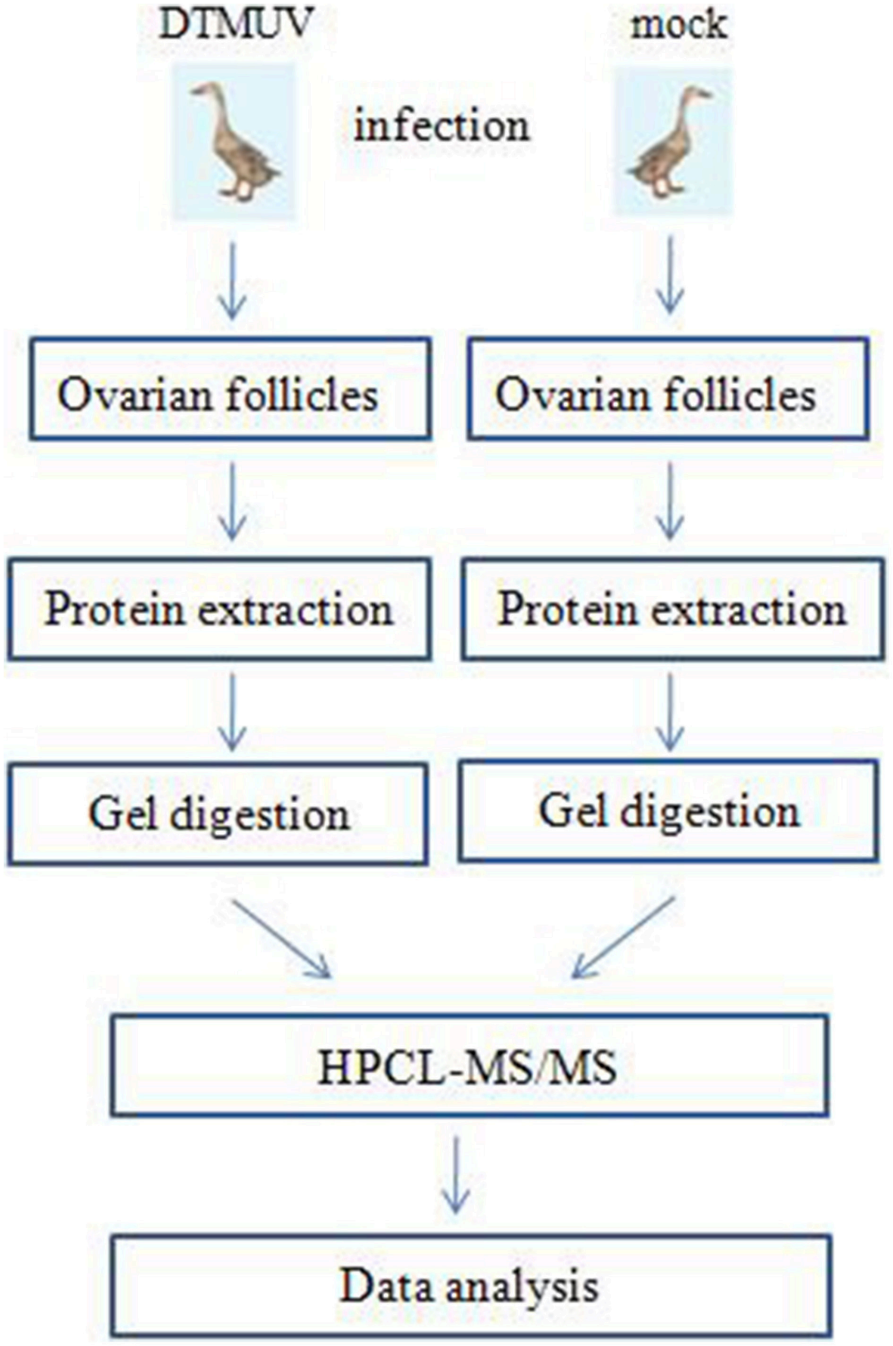
**Label-free profiling flowchart**. DTMUV-infected and mock-infected ovarian follicles were lysed and proteins were extracted. Following tryptic digestion, purified peptides were characterized by LC-MS/MS and subsequent relative quantitative expression profiling.

Figure 1 shows the flowchart of the analysis of the ovarian follicles. For the identification of differentially expressed proteins in the ovarian follicles of the DTMUV-infected ducks, two sets of independent biological replicates of the ovarian follicles from the control (mock-infected group) and DTMUV-infected group were used. The lysates from the samples were used for gel-assisted digestion and LC-MS/MS was performed in triplicate. A total of 1092 and 1176 unique peptides corresponding to 188 and 203 distinct proteins were identified from the two sets of biological replicates, respectively, by optimized LC-MS/MS analysis (*p* < 0.05, protein score ≥ 34). Furthermore, 176 proteins from the two sets of biological replicates overlapped and were subsequently adjusted for multiple testing according to the stringent method of Benjamini and Hochberg (Cho et al., 2012).

In addition, the average coefficient of variation obtained for ratios of all proteins from two biological replicates (three analytical replicates) for both control and TMUV-infected ovarian follicles were determined to be 14% (corresponding to 1.2-fold change). The cutoff criteria considered for the differentially expressed proteins were set with an adjusted *p* value (Benjamini and Hochberg (BH) *p* value) of < 0.05 and a ratio of >2-fold difference. Finally, 131 proteins among the quantified 176 proteins fulfilled the stringent cutoff criteria, of which 53 proteins were up regulated and 78 proteins were down regulated in the DTMUV-infected group. A list of partial regulated proteins is shown in Table 3. Table S1 (Supplemental Material) presents the entire list of proteins, whose expression was modified and the corresponding list of up and down regulated proteins. Protein ratios were presented as mock-infected/TMUV-infected.

**Table 3:** **List of differentially expressed proteins (53 up-regulated and 78 down-regulated) in TMUV-infected ovarian follicles versus mock-infected**.

**Protein name**	**Accession No**.	**Molecular mass (kDa)**	**Sequence Coverage (%)^C^**	**No. of peptide matched^N^**	**(mock /DTMUV)**	
					**Average volume ratio^R^**	***p* value**
Very low density apolipoprotein II	gi|115607579	12.065	46.2	4	34.75	5.16E-15
Proline synthase co-transcribed bacterial homolog protein	gi|514774059	22.642	13.9	2	8.46	1.58E-05
Histone deacetylase 2	gi|514704593	54.649	12.4	4	7.58	5.26E-05
Microsomal glutathione s-transferase 3	gi|483501774	15.799	52.4	5	6.95	0.000128126
Filamin-c isoform x1	gi|62087310	69.735	8.5	4	5.02	0.00253425
C-myc-binding protein	gi|483512092	5.0477	38.6	1	4.84	0.00340488
Basement membrane-specific heparan sulfate proteoglycan core partial	gi|483502305	18.903	39.4	5	4.80	0.00362232
Thrombospondin-1	gi|514705477	127.26	21.3	16	4.72	0.0041384
Syntaxin-7	gi|514705015	29.395	20.6	4	4.64	0.00467332
Low quality protein: apolipoprotein b-100	gi|514717155	523	45.3	164	4.63	0.00477406
Serpin b6	gi|514760039	49.101	24.4	7	4.02	0.0135494
dna damage-binding protein 1	gi|514786465	126.04	6	5	3.98	0.0143964
Myosin-11 isoform x1	gi|483514386	229.49	46.6	55	3.74	0.0220366
Fibrillin- partial	gi|483513220	306.91	19.6	35	3.70	0.0236146
Laminin subunit gamma-1	gi|363736407	170	26.4	27	3.65	0.0258139
Eukaryotic translation initiation factor 4h isoform x1	gi|514779532	27.828	27	5	3.64	0.0259663
Myosin-10 isoform x1	gi|483495208	232.72	47.7	6	3.56	0.0302373
Eukaryotic translation initiation factor 4 gamma 3	gi|483502310	184.61	6.6	7	3.48	0.0348488
Laminin subunit beta-1	gi|514706493	189.99	18.5	23	3.47	0.0354601
Nadh dehydrogenase	gi|514718191	12.947	41.4	3	3.45	0.0367794
Proteasome subunit beta type-7	gi|483506999	24.87	14.4	3	3.43	0.0378593
Succinate dehydrogenase	gi|514715909	31.317	29.1	7	3.35	0.0439268
Citrate lyase subunit beta-like mitochondrial	gi|483491953	25.062	11.6	2	0.46	0.0095273
Interferon regulatory factor 3-like	gi|483516224	44.002	13.9	3	0.46	0.0092195
Tubulin beta-6 chain-like	gi|514706747	50.447	52.5	8	0.45	0.00881894
Apolipoprotein a-I	gi|514704994	29.438	60.9	1	0.43	0.00660928
Transmembrane and coiled-coil domain-containing protein 1	gi|541960426	15.66	10.8	1	0.41	0.0050315
2 -5 -oligoadenylate synthase-like protein 2-like	gi|514778315	11.22	50.5	4	0.40	0.00384987
Tripeptidyl-peptidase 1	gi|483498670	45.841	5.8	2	0.39	0.00320554
Interferon-induced protein with tetratricopeptide repeats 5	gi|514797821	55.849	48.3	21	0.38	0.00280381
Deoxycytidylate deaminase	gi|514714762	20.875	11.4	2	0.36	0.00188472
Protein pml-like	gi|483510961	54.841	26.1	7	0.36	0.00159202
Beta-defensin 10	gi|483501691	7.1343	29.4	1	0.33	0.000922591
26s proteasome non-atpase regulatory subunit 3	gi|483499404	45.223	14.1	4	0.31	0.000503065
Carbonic anhydrase 2	gi|514733257	28.717	67.3	13	0.29	0.000310343
Gamma-glutamyl hydrolase	gi|514704928	37.594	11.3	2	0.21	1.51413E-05
Deubiquitinating protein vcip135	gi|514745303	131.7	1.3	1	0.15	4.47E-07
Zona pellucida protein 1	gi|483510245	15.5	51.8	2	0.13	7.39E-08
atp synthase subunit mitochondrial isoform x1	gi|527254323	61.035	38.2	3	0.07	4.79E-12

*R, Protein expression ratio. Calculated by the mean of corresponding peptide intensities detected in mock-infected versus DTMUV-infected ovarian follicles; N, number of peptides detected; C, detected peptide coverage of protein sequence in %; Values are means ± SEMs. Compared with the control group, proteins with a threshold of >2 or, < 0.5-fold and P < 0.05 were considered differentially expressed proteins. Proteins from two independent biological replicate sets from control and TMUV-infected were used for gel-assisted digestion and then triplicate LC-MS/MS analysis*.

**Figure 2:**
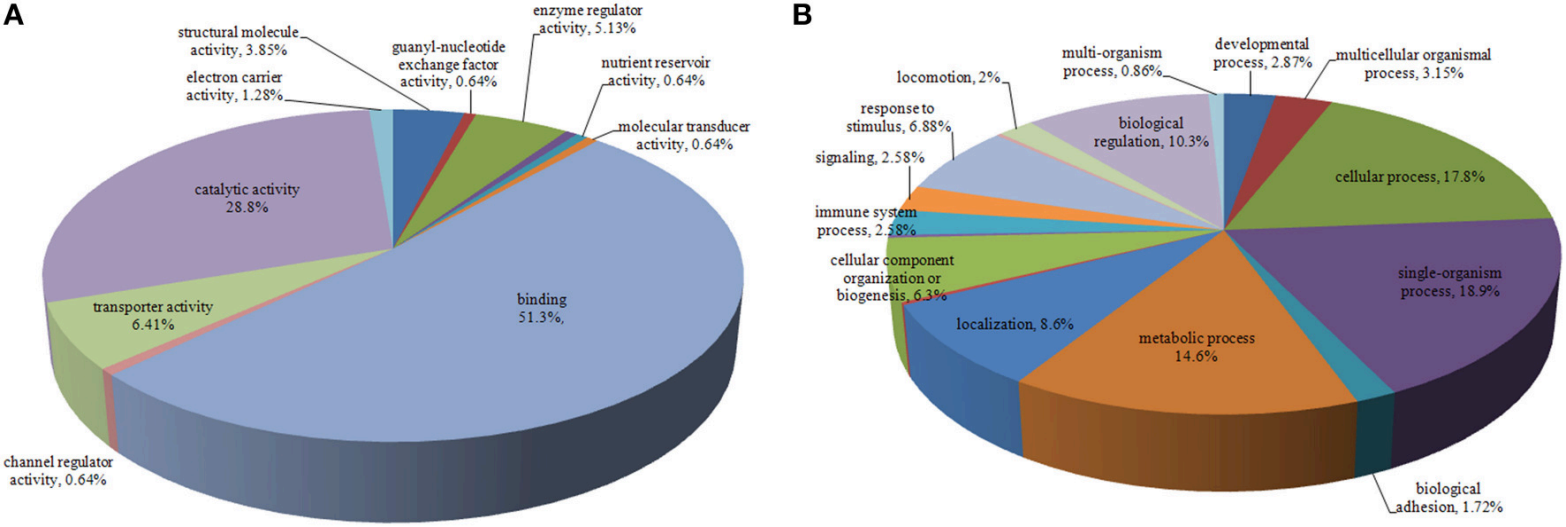
**Pie chart representing differentially expressed proteins identified by label-free LC-MS following DTMUV infected ovarian follicles**. Proteins were classified according to their Molecular Function **(A)**, Biological processes **(B)**.

The identified proteins were categorized according to the GO molecular functional groups. The main molecular functional groups of the proteins that exhibited significantly modified expression levels included catalytic proteins (28.8%), binding proteins (51.3%), transporter proteins (6.41%), enzyme regulator proteins (5.13%), electron carrier proteins (1.28%), structural molecule proteins (3.85%), channel regulator proteins (0.64%), and molecular transducer proteins (0.64%; Figure 2A). The cellular component of these identified proteins indicated that a majority of the shared proteins were localized in the cell (30.3%), organelle (24.8%), membrane (13.2%), macromolecular complex (14.5%), extracellular region (10.7%), and extracellular matrix (2.99%) (Figure S1). Furthermore, the identified proteins were found to be involved in diverse biological processes, such as cellular process, immune system process, biological regulation, signaling, metabolic process, biological adhesion, single-organism process, locomotion, response to stimulus, and multicellular organismal process (Figure 2B).

**Figure 3:**
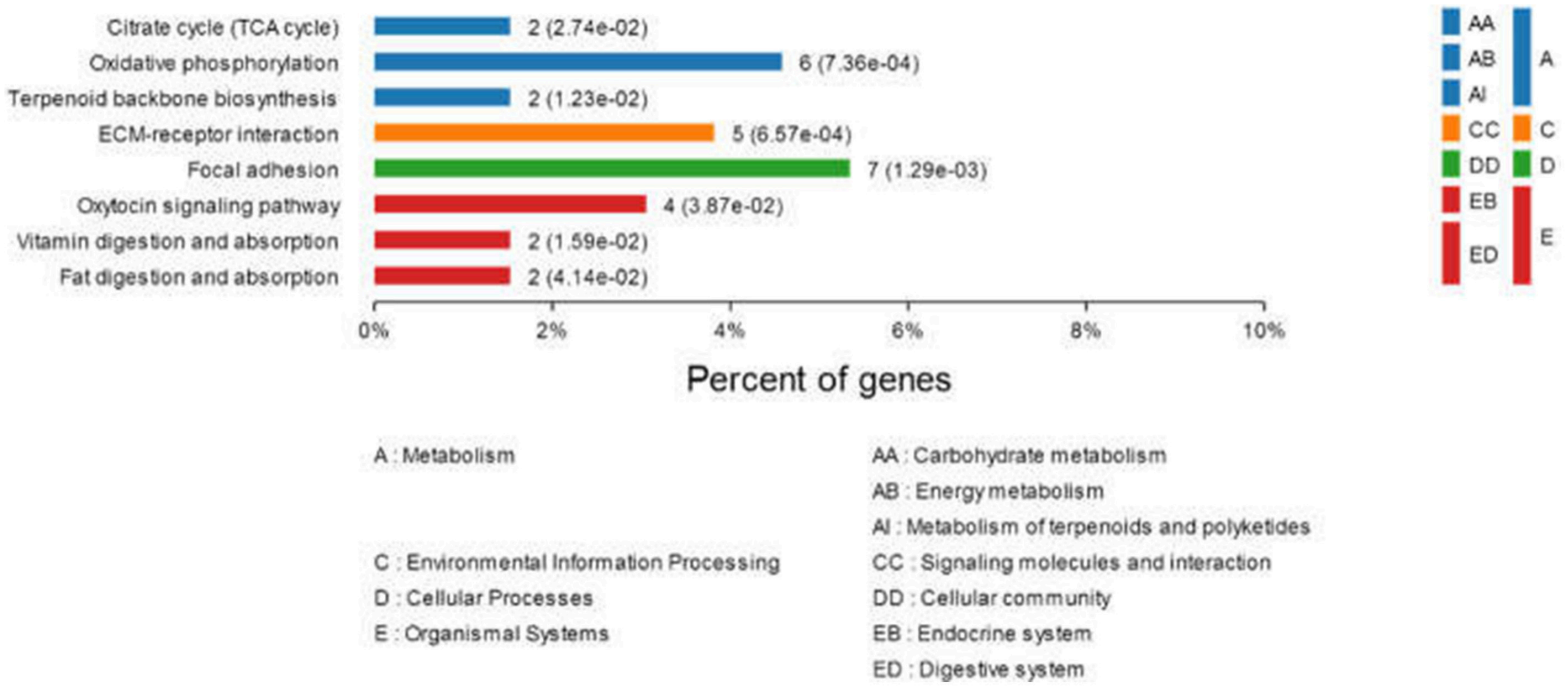
**Comparison of protein expression profiles in duck ovarian follicles infected with DTMUV (***p*** < 0.05)**. Each pathway was statistically significant by a KEGG pathway.

To better understand the interactions between the differentially expressed proteins during DTMUV replication and their possible involvement in viral pathogenesis, networks of inter-relationships of these proteins were constructed using the KEGG pathway program. A series of important pathways, including metabolism (e.g., citrate cycle, oxidative phosphorylation, terpenoid backbone biosynthesis), ECM-receptor interaction, focal adhesion, oxytocin signaling pathway, vitamin digestion, and absorption, and fat digestion and absorption was obtained (Figure 3). In addition, other well-known pathways, including cell adhesion molecules (CAMs), PI3K-Akt signaling pathway, ubiquitin-mediated proteolysis, cAMP signaling pathway, and RIG-I-like receptor signaling pathway were also recorded.

### Analysis of the identified proteins at the transcriptional level

**Figure 4:**
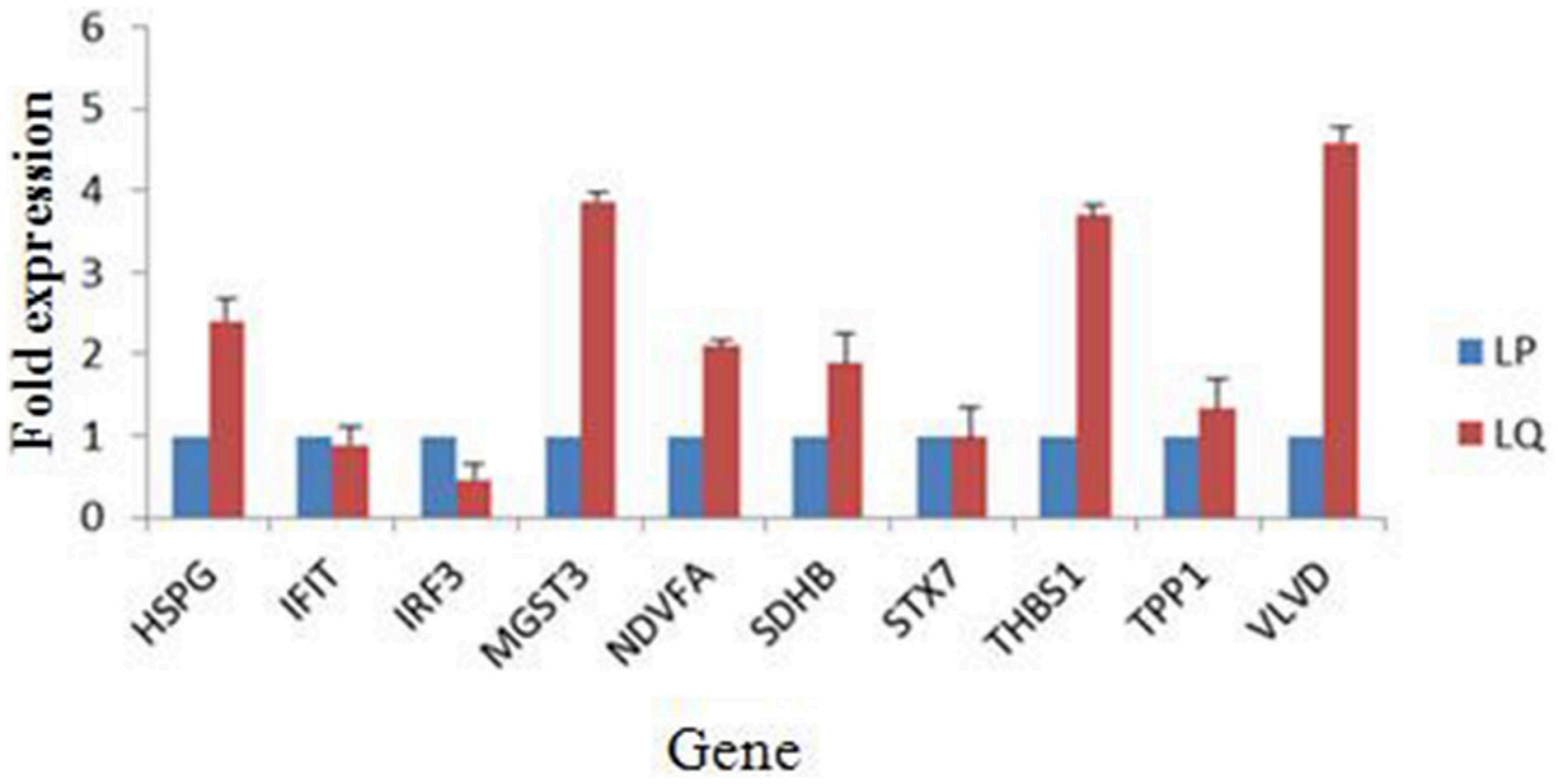
**Transcript alteration of the differentially expressed proteins in DTMUV-infected ovarian follicles**. Total RNA of ovarian follicles (LP, DTMUV- infection; LQ, mock-infection) was measured by real time RT-PCR analysis. Samples were normalized with β-actin gene as a control housekeeping gene. Error bars represent standard deviation. Gene symbols indicating different genes listed as follows. HSPG: heparan sulfate proteoglycan; IFIT, interferon-induced protein with tetratricopeptide repeats; IRF3, interferon regulatory factor 3-like protein; MGST3, microsomal glutathione s-transferase 3; NDVFA, NADH dehydrogenase; SDHB, succinate dehydrogenase; STX7, syntaxin-7; THBS1, thrombospondin-1; TPP1, tripeptidyl-peptidase 1; VLVD, very low density apolipoprotein.

Alterations in the expression of a protein may be owing to a change in its mRNA level. To confirm the results of the proteomic analysis at the mRNA level, the transcriptional alterations in 10 selected proteins were measured by real-time RT-PCR. The β-actin gene was used as the control housekeeping gene. In general, the trends in the change in mRNA abundance were similar to those of their corresponding proteins in the label-free LC-MS (Figure 4). The mRNA level of HSPG, MGST3, NDVFA, SDHB, THBS1, and VLVD were decreased in TMUV-infected ovarian follicles (Figure 4). The mRNA level of IFIT and IRF3 were increased in TMUV-infected ovarian follicles (Figure 4). There was no distinct change in the STX7 at the transcriptional level. This indicated that the fold change in protein expression of STX7 was mainly in the level of translation and post-translation modification. These data except STX7 provide transcriptional information complementary to those differentially expressed proteins detected by label-free LC-MS analysis.

### Confirmation of proteomic data by western blot analysis

**Figure 5:**
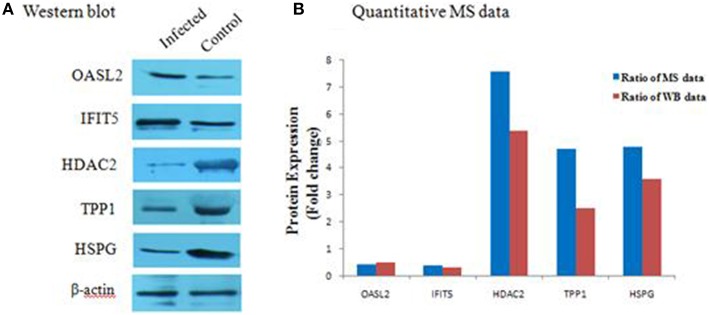
**Western blot analysis of representative proteins identified by LCMS/MS. (A)** differential expression level of proteins, including OASL2, IFIT5, HDAC2, TPP1, and HSPG were detected by western blot analysis. After tissue lysis, equal amount of protein sample (50 μg/lane) were separated by SDS-PAGE. Western blot analysis was then performed using antibodies to these proteins. The β-actin was used as an internal control to normalize the quantitative data. **(B)** results derived from western blot analysis were compared with those from MS analysis.

To further verify the proteins identified by label-free LC-MS methods, five proteins (OASL2, IFIT5, HDAC2, TPP1, and HSPG) were selected for western blot analysis. Equal amounts of tissue lysates from TMUV-infected ovarian follicles and mock-infected ovarian follicles were examined with specific antibodies to these proteins. The data showed in Figure 5 indicated that these proteins were recognized with respective antibodies at different level. Also from Figure 5, it showed that IFIT5 and OASL2 were up regulated in TMUV-infected ovarian follicles, HDAC2, TPP1, and HSPG were down regulated in TMUV-infected ovarian follicles, these results was consistent with the label-free LC-MS analysis. All of above data validated the LC-MS identification of those proteins in DTMUV-infected ovarian follicles that differentially expressed.

## Discussion

The egg-laying performance of layer ducks is the most important economic trait and is regarded as a critic factoring affecting productivity in the layer duck industry. Ovaries play an important role in laying trait. It has been reported that DTMUV affects both meat and laying ducks, significantly reducing egg production ranging from 20 to 60%, and even up to 90%, in the farms of eastern China (Li et al., 2015). At necropsy, the infected ducks displayed severe ovarian hemorrhage, ovaritis, and regression consistently. DTMUV infection may cause dramatic changes in the function and morphology of the host tissues, and even alter the expression of the host cell genes. Recently, increasing attentions have been focused on the interaction between flavivirus and host (Zheng J. et al., 2011). However, there are none studies that have been performed to analyze this interaction using proteomics methods.

Two-dimensional LC-MS/MS enables identification of proteins in a complex mixture by using a combination of HPLC and MS after tryptic proteolysis, and is a powerful method for investigating disease biomarkers (Bartel et al., 2011). In the present study, two-dimensional LC-MS/MS proteomics was used to compare the total proteomes in the DTMUV-infected ovarian follicles with those in the normal ovarian follicle tissues. Quantitative analysis demonstrated dysregulation of 131 proteins in the DTMUV-infected ovarian follicles (53 up regulated and 78 down regulated proteins). The differentially expressed proteins were found to be involved in cytoskeleton organization, macromolecular biosynthesis, signal transduction, stress response, ubiquitin-proteasome pathway (UPP), and metabolic enzymes. All of these results indicate that the development of DTMUV in ovarian follicles is a process that involves multiple factors, in which many proteins and pathways are involved and where binding functions are greatly affected by pathogenesis.

### Changes in host cytoskeleton after DTMUV infection

The cytoskeletal network is a cellular scaffold system whose functions include maintenance of cellular shape, enabling cellular migration, cell division, intracellular transport, signaling, and membrane organization. Many viruses, such as herpes viruses (Falke, 1997), human immunodeficiency virus (HIV; Fackler and Krausslich, 2006), mammalian reovirus (Parker et al., 2002), etc. utilize the host cytoskeleton to promote infection. In the present study, β-tubulin, apolipoprotein, transmembrane, and coiled-coil domain-containing protein were up regulated, whereas filamin-c isoform, fibrillin, myosin-10 isoform, myosin-11 isoform, and unconventional myosin-ic were down regulated in the DTMUV-infected group.

β-tubulin is a microtubule-associated protein, which plays important roles in the lifecycle of viruses. Changes in β-tubulin have been detected in SARS-CoV22 (Jiang et al., 2005) and infectious bursal disease virus (Zheng et al., 2008). Fan et al. (2012) indicated that the β-tubulin level was down regulated in infected PK-15 cells, and speculated that the β-tubulin networks collapse and disperse in host cells leading to an unstable cytoskeletal structure and release of viral particles from the infected cells. Myosins are a large superfamily of motor proteins that are involved in movement along actin filaments, development of myriad cells, targeted organelle transport, endocytosis, chemotaxis, cytokinesis, and signal transduction (Li and Yang, 2015). Some viral proteins can interact with the cytoskeletal transport machinery, such as actin-binding proteins, and induce rearrangements of cytoskeletal filaments so that they can utilize them as tracks or push them aside when they represent barriers (Radtke et al., 2006). In the present study, several actin-binding proteins, such as myosin-10 isoform, were found to be down regulated in the DTMUV-infected group, suggesting that DTMUV may also manipulate the host cytoskeletal network for its own infectious processes and replication. Nevertheless, the roles of these cytoskeletal proteins in ducks after DTMUV infection should be further investigated.

### Energy production and metabolism

Viral replication requires energy and macromolecular precursors derived from the metabolic network of the host. In the present study, the abundance of some proteins associated with carbohydrate, amino acid, and lipid metabolic processes was found to be differentially altered in the DTMUV-infected group. In addition, we identified changes in the abundance of energy metabolism proteins, including β-citrate lyase, deoxycytidylate deaminase, mitochondrial ATP synthase and γ-glutamyl hydrolase, which were up regulated following DTMUV infection. Moreover, down regulation of enzymes, such as proline synthase, serpin b6, NADH dehydrogenase, and succinate dehydrogenase was also observed in the DTMUV-infected group. Mitochondrial ATP synthase catalyzes ATP synthesis using an electrochemical gradient of protons across the inner membrane during oxidative phosphorylation (Devenish et al., 2000). It is constitutively expressed in the inner mitochondrial membrane in normal cells and is involved in the regulation of a variety of cellular functions (Maguire et al., 2006). Abnormal expression of ATP synthase is found to be involved in cell dysfunction and tumorigenesis (López-Ríos et al., 2007). Recently, ATP synthase was reported as a proviral factor that can promote replication of herpes simplex virus-1 (Zheng S. et al., 2011) and H3N2 swine influenza virus (Wu et al., 2013). Thus, the increased ATP synthase expression observed in the present study may possibly contribute to virus replication. ATP citrate lyase catalyzes the generation of acetyl-CoA, a pivotal precursor of fatty acids, from the accumulated citrate in the cytoplasm (Qian et al., 2015). In the flavivirus Hepatitis C virus (HCV), this enzyme is required for cholesterol and fatty acid biosynthesis, and is induced during the initial stages of high-level HCV replication. In addition, more considerable evidence suggests that the cholesterol and fatty-acid-biosynthesis pathways may play a role in HCV replication and infection (Kapadia and Chisari, 2005). Meanwhile, the down-regulation of proteins involved in the glycolytic pathway and TCA cycle suggests that DTMUV infection reduces energy metabolism of the infected cells. This finding is consistent with some previous reports (Fan et al., 2012; Pando-Robles et al., 2014), demonstrating that DTMUV infection promotes changes in mitochondrial bioenergetics, causing an increase in cellular oxygen consumption and decreased efficiency in ATP synthesis (El-Bacha et al., 2007). However, the functional implications of these changes have not been demonstrated and need further research.

### RNA processing and translation machinery

Viruses may inhibit host protein synthesis by targeting multiple steps in the gene expression process via various pathways. For example, the vesicular stomatitis virus M protein inhibits the initiation of the transcription of host genes (Ahmed et al., 2003) and the SARS-CoV spike protein inhibits host cell translation by interaction with eIF3f (Xiao et al., 2008). In the present study, we noted altered expression of several proteins following DTMUV infection, including down-regulation of two eukaryotic translation initiation factors (eIF4 and eIF4H) and c-myc-binding protein and up-regulation of 26s proteasome. The protein eIF4H stimulates protein biosynthesis, ATP hydrolysis, and helicase activity of eIF4A (Bartel et al., 2011). The eukaryotic initiation factor 4 gamma 3 (eIF4G3) is critical for the initiation of protein translation. The eIF4G3 protein is a central player in the initiation of translation since it also contains binding domains for mRNA, eIF4A, and eIF3. It has been reported that many viruses cleave eIF4G3 to stop host protein translation (Marcet-Palacios et al., 2011). For example, rhinoviruses and enteroviruses shut off host protein synthesis by cleaving eIF4G3, resulting in inhibition of cap-dependent translation (Svitkin et al., 1999). Likewise, DTMUV may also play a similar role during its multiplication in host cells.

### The alteration of ubiquitin-proteasome pathway (UPP)

UPP, a major intracellular protein degradation pathway, has recently been implicated in viral infections, including avoidance of host immune surveillance, viral maturation, viral progeny release, efficient viral replication, and reactivation of the virus from latency (Gao and Luo, 2006). In the present study, DTMUV infection induced differential expression of the deubiquitinating protein VCIP135, proteasome 26S subunit, and proteasome subunit beta. It has been demonstrated that VCIP135 possesses deubiquitinating activity, which is essential for p97/p47-mediated Golgi membrane fusion (Zhang and Wang, 2015). The induced levels of VCIP135 observed in the present study might represent a cellular response to counterbalance cellular ubiquitination and degradation, which may facilitate viral progeny release. Nevertheless, further investigations are necessary to determine whether DTMUV possesses another strategy to establish infection.

### Proteins associated with immune response

The abundance of several proteins involved in immune response as well as antigen processing and presentation were also altered following DTMUV infection. The IFIT protein family, comprising IFIT1, IFIT2, IFIT3, and IFIT5, is characterized by multiple repeats of tetratricopeptide repeat helix-turn-helix motifs that mediate a variety of protein-protein interactions (Zhang et al., 2013). These proteins are distinctly induced by type I and II interferons and viruses, which are less abundantly expressed under normal conditions and are mainly concentrated in actin-rich protrusions and apical cell surfaces (Hsu et al., 2013). The IFITs are conserved in mammals, amphibians and fishes, but do not exist in lower animals. In birds, only IFIT5 has been detected (Zhou et al., 2013), and knowledge on its function is very limited. Recently, some reports have indicated that IFIT5 enhances NF-κB activation through increased phosphorylation and activation of IκB kinase (IKK; Yamamoto and Gaynor, 2004). IKK catalyzes stimulus-induced phosphorylation and ubiquitin-mediated degradation of IκB proteins, subsequently activating NF-κB. Further research has suggested that IFIT5 interacts with both IKK and TGF-β-activated kinase 1 (TAK1; Fan et al., 2011). Moreover, IFIT5 facilitates the interaction between TAK1 and IKK that contributes to IKK phosphorylation. Zhang et al. (2013) reported that IFIT5 was significantly induced at both mRNA and protein levels upon Sendai virus infection, and that the host antiviral responses were markedly enhanced or impaired in the presence or absence of IFIT5, respectively. In the present study, we detected up-regulation of IFIT5 following DTMUV infection in the ovarian follicles, however, its role in antiviral process and immune regulation requires further investigation.

The 2′-5′oligoadenylate synthase (OAS) family consists of OAS1, OAS2, OAS3, and OAS-like protein (OASL). The oligomerized OAS protein generates 2′-5′-linked oligoadenylate (2-5A), activating endoribonuclease and RNase L (Pulit-Penaloza et al., 2012). It has been demonstrated that RNase L contributes to the control of early spread of virus by degrading the viral RNA and activating cytoplasmic pattern recognition receptors, including RIG-I and MDA-5 (Zhu et al., 2014). Recently, the OAS family proteins were noted to show RNase-L-dependent antiviral activity against dengue virus, West Nile virus, HCV, and Japanese encephalitis virus (Deo et al., 2014), all of which belong to the family *Flaviviridae*. Thus, the OASL screened in the present study may be associated with differential susceptibility to the clinical outcomes of Tembusu virus infection.

## Conclusions

In summary, we have performed the first analysis of the proteomic changes in duck ovarian follicles during DTMUV infection *in vivo*. Also, we have shown that infection of ovarian follicles induces changes in expression of 131 proteins. Our data revealed significant changes in the proteins associated with energy metabolism, RNA processing and translation machinery, host cytoskeleton, and immune response. The modulation of protein expression found in our study may attribute to the strategy of the virus to overcome host pathways to facilitate survival at the expense of the host. Further, studies are needed to understand the detailed mechanism by which these proteins are induced during viral infection. Virus could make use of the transcription and translation of some host products for their survival, but the detailed mechanism is still unknown. The modulation of protein expression could also be the host's response to the viral infection. It would be interesting in a further study, to determine whether a similar response could occur in cells infected with other viruses or if the observed modulation of protein expression is specifically induce in response to DTMUV infection. Improvements in our knowledge on target organ will be important to decipher the infection process of Tembusu virus in ducks.

## Author contributions

KH and YL designed experiments; KH, DZ, YL, QL, and XH carried out experiments; JY and FA analyzed experimental results; KH wrote the manuscript.

### Conflict of interest statement

The authors declare that the research was conducted in the absence of any commercial or financial relationships that could be construed as a potential conflict of interest.
