# Supplementary material for: Quantitative Proteomic Analysis of Duck Ovarian Follicles Infected with Duck Tembusu Virus by Label-Free LC-MS
Source: Front Microbiol. 2016 Mar 31;7:463. doi: 10.3389/fmicb.2016.00463 (PMC4815560; doi:10.3389/fmicb.2016.00463)
Supplement: Supplementary file 1 [file Table1.DOCX]

| **Protein name** | **Accession No.** | **Molecular mass (kDa)** | **Sequence Coverage (%)C** | **No. of peptide matched** | **( mock /DTMUV)** | |
| --- | --- | --- | --- | --- | --- | --- |
|  |  |  |  |  | **Average volume ratioR** | **p value** |
| vitellogenin-1-like | gi\|483523958 | 204.85 | 59.4 | 93 | 75.33 | 1.98E-22 |
| vitellogenin-2-like | gi\|483509788 | 184.1 | 60.5 | 79 | 38.17 | 7.93E-16 |
| riboflavin-binding protein | gi\|514715525 | 27.48 | 28 | 5 | 35.64 | 3.14E-15 |
| prolyl 4-hydroxylase subunit alpha-2 isoform x1 | gi\|514766085 | 60.128 | 10.3 | 3 | 34.87 | 4.84E-15 |
| very low density apolipoprotein Ⅱ | gi\|115607579 | 12.065 | 46.2 | 4 | 34.75 | 5.16E-15 |
| vitellogenin-2-like | gi\|514757582 | 202.6 | 66.2 | 98 | 33.08 | 1.35E-14 |
| proline synthase co-transcribed bacterial homolog protein | gi\|514774059 | 22.642 | 13.9 | 2 | 8.46 | 1.58E-05 |
| histone deacetylase 2 | gi\|514704593 | 54.649 | 12.4 | 4 | 7.58 | 5.26E-05 |
| cytochrome b-c1 complex subunit mitochondrial-like | gi\|483507487 | 22.557 | 34.8 | 4 | 6.96 | 0.000126336 |
| microsomal glutathione s-transferase 3 | gi\|483501774 | 15.799 | 52.4 | 5 | 6.95 | 0.000128126 |
| mhc class i antigen alpha chain | gi\|34148112 | 37.693 | 28.7 | 1 | 6.76 | 0.000169156 |
| golgi-specific brefeldin a-resistance guanine nucleotide exchange factor 1 isoform x2 | gi\|514769814 | 207.58 | 0.6 | 1 | 6.76 | 0.000170211 |
| tens1_human ame: full=tensin-1 | gi\|66529407 | 176.46 | 7.7 | 7 | 6.48 | 0.000255572 |
| clip-associating protein 1 isoform x1 | gi\|483515580 | 166.92 | 1.6 | 2 | 5.99 | 0.000540347 |
| leucine-rich repeat-containing protein 15 | gi\|541983807 | 47.2 | 19.9 | 5 | 5.86 | 0.000662913 |
| collagen alpha-2 chain | gi\|514708854 | 128.93 | 12.2 | 12 | 5.81 | 0.000718149 |
| tbc1 domain family member 15 | gi\|514766481 | 77.091 | 5.2 | 3 | 5.69 | 0.000856539 |
| protein nynrin | gi\|527252769 | 87.09 | 36.5 | 6 | 5.51 | 0.00113444 |
| calcium calmodulin-dependent protein kinase type ii subunit delta isoform 1 | gi\|557299007 | 45.504 | 16.6 | 4 | 5.40 | 0.00135995 |
| acyl-protein thioesterase 2 | gi\|514717668 | 16.289 | 10.5 | 2 | 5.18 | 0.00195103 |
| dynamin-1-like protein isoform x4 | gi\|527252301 | 80.334 | 19.8 | 10 | 5.17 | 0.00196865 |
| phosphomevalonate partial | gi\|483500793 | 16.957 | 23.8 | 3 | 5.04 | 0.00243956 |
| filamin-c isoform x1 | gi\|62087310 | 69.735 | 8.5 | 4 | 5.02 | 0.00253425 |
| c-binding partial | gi\|541970041 | 74.598 | 8.9 | 4 | 4.99 | 0.00264503 |
| c-myc-binding protein | gi\|483512092 | 5.0477 | 38.6 | 1 | 4.84 | 0.00340488 |
| cadherin- partial | gi\|483512428 | 76.846 | 10.3 | 4 | 4.84 | 0.00340635 |
| basement membrane-specific heparan sulfate proteoglycan core partial | gi\|483502305 | 18.903 | 39.4 | 5 | 4.80 | 0.00362232 |
| tyrosine-protein phosphatase auxilin | gi\|483487695 | 100.9 | 2.4 | 2 | 4.79 | 0.00369095 |
| thrombospondin-1 | gi\|514705477 | 127.26 | 21.3 | 16 | 4.72 | 0.0041384 |
| protein disulfide-isomerase a5 | gi\|514737565 | 59.946 | 20.5 | 6 | 4.66 | 0.00453853 |
| inosine-5 -monophosphate dehydrogenase 2 | gi\|483503591 | 47.289 | 23.2 | 6 | 4.65 | 0.00463263 |
| vesicle-associated membrane protein 7-like isoform x1 | gi\|514713186 | 23.924 | 23.5 | 4 | 4.64 | 0.00467231 |
| syntaxin-7 | gi\|514705015 | 29.395 | 20.6 | 4 | 4.64 | 0.00467332 |
| low quality protein: apolipoprotein b-100 | gi\|514717155 | 523 | 45.3 | 164 | 4.63 | 0.00477406 |
| lipoamide acyltransferase component of branched-chain alpha-keto acid dehydrogenase mitochondrial | gi\|514742845 | 52.303 | 8 | 3 | 4.47 | 0.00626641 |
| peptidyl-prolyl cis-trans isomerase nima-interacting 1 | gi\|514740522 | 17.883 | 34 | 4 | 4.46 | 0.00637473 |
| protein fam98b | gi\|514705487 | 41.231 | 14.3 | 3 | 4.30 | 0.00829714 |
| polymerase i and transcript release factor | gi\|514713564 | 41.282 | 15.2 | 4 | 4.19 | 0.0101536 |
| pdz and lim domain protein 7 | gi\|543360400 | 63.836 | 19.1 | 7 | 4.16 | 0.0105246 |
| nadh dehydrogenase | gi\|514788777 | 20.122 | 41.9 | 5 | 4.10 | 0.0118495 |
| c-binding | gi\|483494768 | 35.852 | 15 | 3 | 4.06 | 0.0125026 |
| serpin b6 | gi\|514760039 | 49.101 | 24.4 | 7 | 4.02 | 0.0135494 |
| dna damage-binding protein 1 | gi\|514786465 | 126.04 | 6 | 5 | 3.98 | 0.0143964 |
| sequestosome-1 isoform x1 | gi\|514766059 | 44.374 | 21.8 | 5 | 3.89 | 0.0169943 |
| PREDICTED: uncharacterized protein LOC101800106 isoform X5 | gi\|514721728 | 84.431 | 3.7 | 2 | 3.86 | 0.0176814 |
| guanine nucleotide-binding protein g subunit alpha isoform x1 | gi\|525010110 | 34.656 | 23.3 | 3 | 3.85 | 0.0181863 |
| collagen alpha-1 chain | gi\|471380401 | 87.848 | 9.9 | 7 | 3.82 | 0.0191248 |
| unconventional myosin-ic | gi\|514769047 | 122.53 | 17.4 | 13 | 3.77 | 0.0209592 |
| epidermal growth factor receptor substrate partial | gi\|483503096 | 98.674 | 4.7 | 3 | 3.76 | 0.0211067 |
| myosin-11 isoform x1 | gi\|483514386 | 229.49 | 46.6 | 55 | 3.74 | 0.0220366 |
| cysteine and glycine-rich protein 1 isoform x1 | gi\|483520015 | 20.555 | 53.4 | 8 | 3.74 | 0.0221041 |
| fibrillin- partial | gi\|483513220 | 306.91 | 19.6 | 35 | 3.70 | 0.0236146 |
| c-1-tetrahydrofolate cytoplasmic | gi\|514759177 | 100.91 | 7.2 | 6 | 3.69 | 0.0239968 |
| light chain 12 | gi\|483514962 | 19.391 | 60.4 | 5 | 3.67 | 0.0249051 |
| laminin subunit gamma-1 | gi\|363736407 | 170 | 26.4 | 27 | 3.65 | 0.0258139 |
| eukaryotic translation initiation factor 4h isoform x1 | gi\|514779532 | 27.828 | 27 | 5 | 3.64 | 0.0259663 |
| neuroblast differentiation-associated protein ahnak-like | gi\|514785002 | 589.53 | 31.8 | 49 | 3.62 | 0.0270139 |
| low-density lipoprotein receptor-related protein 1-like | gi\|514764486 | 504.64 | 5.1 | 15 | 3.60 | 0.0282812 |
| phosphatidylcholine-sterol acyltransferase-like | gi\|514722922 | 51.469 | 13.3 | 4 | 3.57 | 0.0295308 |
| acetolactate synthase-like protein | gi\|483503698 | 33.11 | 22.7 | 1 | 3.56 | 0.0299688 |
| myosin-10 isoform x1 | gi\|483495208 | 232.72 | 47.7 | 6 | 3.56 | 0.0302373 |
| protein mrp-126-like | gi\|514712023 | 13.945 | 25.2 | 3 | 3.52 | 0.0322128 |
| sorbin and sh3 domain-containing protein 2 isoform x2 | gi\|514703820 | 125.32 | 2.2 | 2 | 3.50 | 0.0336603 |
| eukaryotic translation initiation factor 4 gamma 3 | gi\|483502310 | 184.61 | 6.6 | 7 | 3.48 | 0.0348488 |
| laminin subunit beta-1 | gi\|514706493 | 189.99 | 18.5 | 23 | 3.47 | 0.0354601 |
| sterol o-acyltransferase partial | gi\|514745007 | 58.575 | 17.3 | 7 | 3.47 | 0.035494 |
| mhc class I antigen alpha chain | gi\|61661528 | 38.529 | 27.3 | 1 | 3.46 | 0.0360317 |
| mitochondrial inner membrane protein | gi\|514795742 | 17.644 | 7.3 | 1 | 3.45 | 0.0365866 |
| nadh dehydrogenase | gi\|514718191 | 12.947 | 41.4 | 3 | 3.45 | 0.0367794 |
| prenylcysteine oxidase-like | gi\|514784292 | 50.982 | 23.5 | 7 | 3.44 | 0.0370228 |
| protein phosphatase 1 regulatory subunit 12a isoform x1 | gi\|483523416 | 106.49 | 4.1 | 2 | 3.44 | 0.0372021 |
| proteasome subunit beta type-7 | gi\|483506999 | 24.87 | 14.4 | 3 | 3.43 | 0.0378593 |
| lipoma-preferred partner homolog | gi\|326925972 | 66.155 | 31.2 | 11 | 3.38 | 0.0410345 |
| fibulin- partial | gi\|483524209 | 77.788 | 25.8 | 10 | 3.38 | 0.0413819 |
| succinate dehydrogenase | gi\|514715909 | 31.317 | 29.1 | 7 | 3.35 | 0.0439268 |
| protein nynrin-like | gi\|483508496 | 145.99 | 47.7 | 36 | 3.33 | 0.0452787 |
| alpha-tectorin-like isoform x4 | gi\|483494770 | 30.233 | 24.6 | 5 | 3.31 | 0.0467319 |
| complement c4 | gi\|514785453 | 178.37 | 15 | 15 | 0.49 | 0.0153482 |
| coiled-coil-helix-coiled-coil-helix domain-containing protein mitochondrial | gi\|483523608 | 10.029 | 26.2 | 2 | 0.49 | 0.0153364 |
| synaptophysin-like protein 1 | gi\|514706437 | 27.331 | 20.6 | 4 | 0.48 | 0.0121481 |
| protein pml-like isoform x1 | gi\|513200871 | 11.612 | 15.5 | 1 | 0.48 | 0.0120288 |
| fetuin-b | gi\|514767542 | 44.21 | 46.7 | 10 | 0.47 | 0.0105217 |
| lish domain and heat repeat-containing protein kiaa1468 homolog | gi\|514752528 | 117.75 | 4.4 | 2 | 0.46 | 0.010028 |
| citrate lyase subunit beta-like mitochondrial | gi\|483491953 | 25.062 | 11.6 | 2 | 0.46 | 0.0095273 |
| interferon regulatory factor 3-like | gi\|483516224 | 44.002 | 13.9 | 3 | 0.46 | 0.0092195 |
| hematological and neurological expressed 1-like protein | gi\|514704170 | 23.52 | 24.8 | 3 | 0.46 | 0.00910089 |
| tubulin beta-6 chain-like | gi\|514706747 | 50.447 | 52.5 | 8 | 0.45 | 0.00881894 |
| general transcription factor iih subunit 1 | gi\|514784079 | 62.072 | 4.2 | 2 | 0.45 | 0.0080274 |
| sialidase-2 isoform x1 | gi\|483498766 | 45.656 | 7.2 | 2 | 0.44 | 0.0072727 |
| histone h1-like | gi\|121943 | 21.831 | 14.7 | 3 | 0.44 | 0.00719436 |
| apolipoprotein a-I | gi\|514704994 | 29.438 | 60.9 | 1 | 0.43 | 0.00660928 |
| pituitary tumor-transforming gene 1 protein-interacting protein | gi\|514723369 | 14.997 | 14.8 | 2 | 0.43 | 0.00584762 |
| steroid 17-alpha-hydroxylase lyase-like | gi\|514772677 | 42.179 | 44.9 | 10 | 0.41 | 0.0044025 |
| transmembrane and coiled-coil domain-containing protein 1 | gi\|541960426 | 15.66 | 10.8 | 1 | 0.41 | 0.0050315 |
| steroid 17-alpha-hydroxylase lyase-like | gi\|514772677 | 42.179 | 44.9 | 10 | 0.41 | 0.0044025 |
| retinol-binding protein 5 | gi\|542154483 | 15.272 | 25.2 | 3 | 0.41 | 0.00435376 |
| hydroxyacylglutathione mitochondrial isoform x1 | gi\|483508368 | 32.126 | 17.1 | 4 | 0.41 | 0.00430096 |
| 2 -5 -oligoadenylate synthase-like protein 2-like | gi\|514778315 | 11.22 | 50.5 | 4 | 0.40 | 0.00384987 |
| tripeptidyl-peptidase 1 | gi\|483498670 | 45.841 | 5.8 | 2 | 0.39 | 0.00320554 |
| mhc class i antigen | gi\|61661526 | 38.539 | 40.4 | 4 | 0.39 | 0.00314209 |
| interferon-induced protein with tetratricopeptide repeats 5 | gi\|514797821 | 55.849 | 48.3 | 21 | 0.38 | 0.00280381 |
| peroxisomal trans-2-enoyl- reductase | gi\|483495832 | 28.555 | 13.5 | 3 | 0.38 | 0.00262468 |
| deoxycytidylate deaminase | gi\|514714762 | 20.875 | 11.4 | 2 | 0.36 | 0.00188472 |
| protein pml-like | gi\|483510961 | 54.841 | 26.1 | 7 | 0.36 | 0.00159202 |
| vam6 vps39-like protein | gi\|71894867 | 98.429 | 4.1 | 3 | 0.36 | 0.00158474 |
| alpha-1-antiproteinase 2-like | gi\|514754079 | 48.307 | 16.5 | 5 | 0.35 | 0.00142111 |
| transporter associated with antigen processing 1 | gi\|169730378 | 60.136 | 19.9 | 2 | 0.34 | 0.00125459 |
| low quality protein: adenosine 3 -phospho 5 -phosphosulfate transporter 1 | gi\|514729408 | 47.87 | 6.2 | 2 | 0.34 | 0.00117753 |
| ump-cmp kinase mitochondrial | gi\|483514318 | 16.432 | 16.8 | 2 | 0.33 | 0.000990123 |
| beta-defensin 10 | gi\|483501691 | 7.1343 | 29.4 | 1 | 0.33 | 0.000922591 |
| zona pellucida protein 1 | gi\|146411660 | 100.64 | 7.2 | 4 | 0.32 | 0.000702728 |
| succinate dehydrogenase cytochrome b560 mitochondrial | gi\|483497625 | 24.162 | 10.7 | 2 | 0.31 | 0.000558784 |
| 26s proteasome non-atpase regulatory subunit 3 | gi\|483499404 | 45.223 | 14.1 | 4 | 0.31 | 0.000503065 |
| ig lambda chain v-1 partial | gi\|483497631 | 10.69 | 24 | 2 | 0.30 | 0.000452748 |
| serum amyloid a | gi\|2507357 | 13.873 | 18.9 | 2 | 0.30 | 0.000417165 |
| lactadherin isoform x2 | gi\|543726091 | 51.28 | 7.9 | 3 | 0.29 | 0.000348379 |
| carbonic anhydrase 2 | gi\|514733257 | 28.717 | 67.3 | 13 | 0.29 | 0.000310343 |
| plasmalemma vesicle-associated protein | gi\|483491812 | 46.169 | 9.5 | 3 | 0.27 | 0.000154095 |
| low quality protein: epiplakin 1 | gi\|483504778 | 66.608 | 13.9 | 2 | 0.22 | 2.11122E-05 |
| gamma-glutamyl hydrolase | gi\|514704928 | 37.594 | 11.3 | 2 | 0.21 | 1.51413E-05 |
| mitochondrial-processing peptidase subunit partial | gi\|514759335 | 57.001 | 5.5 | 2 | 0.20 | 1.20724E-05 |
| deubiquitinating protein vcip135 | gi\|514745303 | 131.7 | 1.3 | 1 | 0.15 | 4.47E-07 |
| alpha-1-antiproteinase 2-like | gi\|514754086 | 46.848 | 23.9 | 5 | 0.15 | 3.09E-07 |
| zona pellucida protein 1 | gi\|483510245 | 15.5 | 51.8 | 2 | 0.13 | 7.39E-08 |
| interferon alpha-inducible protein 27-like protein 2b-like | gi\|483502446 | 10.331 | 69.8 | 5 | 0.12 | 3.86E-08 |
| dystonin isoform x3 | gi\|483513364 | 845.25 | 0.6 | 4 | 0.12 | 3.10E-08 |
| zona pellucida c | gi\|514753582 | 40.355 | 54.5 | 15 | 0.10 | 2.44E-09 |
| atp synthase subunit mitochondrial isoform x1 | gi\|527254323 | 61.035 | 38.2 | 3 | 0.07 | 4.79E-12 |
| yth domain family protein 1 | gi\|53127426 | 61.178 | 8.4 | 1 | 0.06 | 2.18717E-12 |
| lipopolysaccharide-responsive and beige-like anchor protein | gi\|483519035 | 316.27 | 0.7 | 2 | 0.05 | 7.786E-14 |
| low quality protein: nesprin-1 | gi\|483506581 | 1010.4 | 0.2 | 2 | 0.03 | 2.8757E-16 |
