# Supplementary figures and images for: Quantitative Proteomic Analysis of Duck Ovarian Follicles Infected with Duck Tembusu Virus by Label-Free LC-MS
Source: Front Microbiol. 2016 Mar 31;7:463. doi: 10.3389/fmicb.2016.00463 (PMC4815560; doi:10.3389/fmicb.2016.00463)

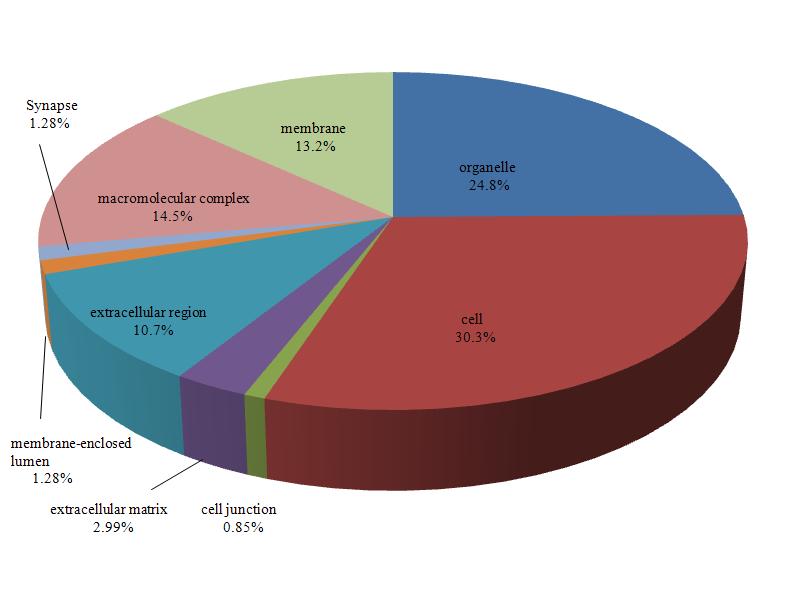

Supplement: Supplementary file 2 [file Image1.JPEG]
